# DNA methylation on C5-Cytosine and N6-Adenine in the *Bursaphelenchus xylophilus* genome

**DOI:** 10.1186/s12864-023-09783-7

**Published:** 2023-11-07

**Authors:** Zhenkai Liu, Yongxia Li, Xingyao Zhang

**Affiliations:** 1 Key Laboratory of Forest Protection of National Forestry and Grassland Administration, Ecology and Nature Conservation Institute, Chinese Academy of Forestry, Beijing, 100091 PR China; 2 Co-Innovation Center for Sustainable Forestry in Southern China, Nanjing Forestry University, Nanjing, 210037 Jiangsu PR China

**Keywords:** Pine Wood Nematode, DNA methylation, 5mC, 6mA

## Abstract

**Background:**

The pinewood nematode is the causal agent of the pine wilt disease, which causes severe ecological and economic losses in coniferous forests. The invasion of pine wood nematode has undergone various rapid adaptations to a wide range of temperatures and to new hosts and vector insects. DNA methylation may play crucial roles in the rapid adaptation of PWN during invasion. However, whether the PWN genome contins functional DNA modifications remains elusive.

**Results:**

Here, we detected the extensive presence of 5-methylcytosine (5mC) and N6-methyladenine (6mA) in the *B. xylophilus* genome, with low methylation levels at most positions. Cytosines were methylated in the CpG, CHG. and CHH sequence contexts, with the lowest methylation levels at CpG sites. The methylation levels of CpG and 6mA in gene regions showed opposite trends. The changes in the abundance of 5mC and 6mA showed the same trends in response to temperature change, but opposite trends during development. Sequence and phylogenetic analyses showed that the proteins BxDAMT and BxNMAD have typical characteristics of a methylase and demethylase, respectively, and are conserved among species.

**Conclusions:**

These findings shed light on the epigenetic modifications present in the genome of PWN, and will improve our understanding of its invasiveness and evolution.

**Supplementary Information:**

The online version contains supplementary material available at 10.1186/s12864-023-09783-7.

## Background

Pinewood nematode (PWN), *Bursaphelenchus xylophilus* (Nematode: Aphelenchoididae), is the causal agent of the pine wilt disease, which causes severe ecological and economic losses in coniferous forests [1].

As a globally destructive plant parasitic nematode, *B. xylophilus* is native to North America and is now distributed in Japan, China, Korea, Portugal, and Spain [2]. In different countries and regions, PWN can adapt to the local environment, host, and vector insects, thus causing serious damage to pine forests. Damage from PWN is, particularly evident in China. Infections of PWN were first detected in the subtropical region of China in 1982 [3]. By 2022, PWN had been detected in 731 counties of 19 provinces, with the northeast region (Jilin Province) being seriously affected (State Forestry Administration of the People’s Republic of China, 2022). During the process of invasion, the host range of PWN has expanded to include *Pinus massoniana* and *Pinus kesiya* var. *langbianensis* in the subtropical zone, *Pinus thunbergii* and *Pinus armandii* in the warm temperate zone, *Pinus tabulaeformis* and *Pinus koraiensis* in the middle temperate zone, and *Larix kaempferi* and *Larix olgensis* in the cold temperate zone. Meanwhile, in addition to *Monochamus alternatus*, new insect vectors are constantly appearing such as *Monochamus salturius* and *Monochamus sutor*. Thus, during a period of 40 years, PWN has undergone various rapid adaptations to a wide range of temperatures and to new hosts and vector insects.

Biological invasions represent one of the greatest threats to ecosystems and biodiversity, but they also provide new examples to investigate rapid adaptation and evolution [4]. Compared with the ability to adapt to the external environment through mutation, genetic drift, and genetic selection in long-term adaptation scenarios, epigenetic modification allows for the active adaptation of organisms to changing environments, thereby increasing the organism’s evolutionary potential [5, 6]. As the most well-studied type of epigenetic modification, DNA methylation plays a crucial role in rapid local adaptation to novel environments in numerous species [7, 8]. There are two main forms of DNA methylation in eukaryotes: 5-methylcytosine (5mC) and N6-methyladenine (6mA).

The 5mC modification has been well established as an epigenomic mark in eukaryotes. In multicellular eukaryotes, the 5mC modification plays an essential role in the regulation of various genes during multiple developmental and physiological processes, including gene expression, genome maintenance, and parental imprinting [9]. The results of previous studies suggested that DNA methylation does not occur in nematodes because no 5mC was detected in the model species *Caenorhabditis elegans*, and because *C. elegans* does not contain homologs of the enzymes that add methyl moieties to cytosine, that is, DNA (cytosine-5-)-methyltransferase 1 (DNMT1) or DNMT3. However, with the recent advent of more sensitive detection techniques, two types of DNA methylation have been detected in nematodes [10–12]. The first type, 5mC, plays an important role as a dominant epigenetic mark in nematodes such as the animal parasitic nematode *Trichinella spiralis* [11]. In most metazoans, including nematodes, the DNMTs identified to date are homologs of DNMT1, DNMT2, or DNMT3. Of them, DNMT1 and DNMT3 have methyltransferase activity and catalyze *de novo* methylation [13].

The other type of DNA methylation detected in nematodes is 6mA, which was discovered in *C. elegans* along with the DNA demethylase, NMAD-1, and the putative DNA-adenine methyltransferase, DAMT-1 [12]. In addition to nematodes, other organisms such as *Chlamydomonas*, *Drosophila*, *Arabidopsis*, rice, and mouse also have the 6mA modification in their genomes, raising the possibility that 6mA may be more prevalent in eukaryote genomes [14–18]. The results of those studies shed light on the diverse functions of 6mA in eukaryotes, including the regulation of gene expression, transposon mobility, and cross-talk with histone modifications, as well as serving as a marker to distinguish invading foreign DNA from host DNA in prokaryotes [17, 19].

DNA methylation may play crucial roles in the rapid adaptation of PWN during invasion. However, whether the PWN genome has functional DNA modifications remained elusive. In this study, we detected the extensive presence of the DNA modifications 5mC and 6mA in the *B. xylophilus* genome, with low methylation levels at most positions. We also found that 5mC and 6mA appeared to occupy distinct regions of the genome, suggesting that they have distinct genomic functions. Changes in the percentage of 5mC and 6mA showed the same trend in response to temperature change, but showed opposite trends during development. The results provide insights into the adaptation and evolution of PWN during its successful invasion in China. Such information is crucial for developing strategies to prevent and manage PWN invasions.

## Methods

### Nematode source and culture conditions

The *B. xylophilus* strain Nxy 61 was isolated from infected *P. massoniana* from Zhejiang, China, and preserved at the Pine Wood Nematode Laboratory of the Chinese Academy of Forestry, Beijing, China. The Nxy 61 PWN strain was cultured on potato dextrose agar with *Botrytis cinerea* at 25 °C in the dark.

### ELISA to detected DNA methylation in the B. ***xylophilus*** genome

Global 5mC and 6mA levels in *B. xylophilus* genomic DNA were detected using the MethylFlash Methylated DNA 5-mC Quantification Kit and the MethylFlash m6A DNA Methylation ELISA Kit (Epigentek, Farmingdale, NY, USA) according to the manufacturer’s protocols. In brief, DNA was extracted from the nematodes using a DNA Isolation Kit (TIANGEN, Beijing, China) and RNA was removed using RNase A (TIANGEN). The extracted DNA was separated on a 1% agarose gel and quantified using a NanoDrop spectrophotometer (Thermo Scientific, Waltham, MA, USA). Then, 200 ng purified DNA per sample was subjected to 5mC and 6mA detection assays using the MethylFlash™ methylated DNA quantification kits described above (Epigentek). The levels of 5mC and 6mA in different DNA samples were quantified according to the manufacturer’s instructions, with both negative and positive controls included in the analyses.

### Detection of DNA methylation in ***B. xylophilus*** by HPLC-MS/MS

The extracted genomic DNA was first digested using the restriction enzyme *Dpn*I followed by ultrafiltration to remove any contaminating bacterial DNA. Then, the DNA (dissolved in water) was denatured by heating at 95 °C for 5 min and then chilled on ice for 2 min. After adding S1 nuclease buffer, alkaline phosphatase, and deoxyribonuclease, the mixture was incubated at 37 °C. After the DNA was completely digested into nucleosides, the mixture was extracted with chloroform. The resulting aqueous layer was collected, reconstituted in water, and then analyzed by ultra-high performance liquid chromatography-multiple reaction monitoring-multi-stage/mass spectrometry (UPLC-MRM-MS/MS).

### Detection of DNA methylation in ***B. xylophilus*** using Oxford Nanopore technologies

Next, we screened for genome-wide DNA methylation (5mC & 6mA) in PWN using Oxford Nanopore Technologies (ONT) methods [20]. Standard protocols were used for the ONT procedures, including sample quality detection, library construction, library quality detection, and library sequencing. Total genomic DNA was isolated using the TIANamp DNA kit (TIANGEN). The purity, concentration, and integrity of extracted genomic DNA were tested using a Nanodrop spectrophotometer (Thermo Fisher Scientific) and a Qubit fluorometer (Invitrogen, Carlsbad, CA, USA), and by 0.5% agarose gel electrophoresis.

The base-called FAST5 files that were generated using ONT were modified to fastq format using Guppy for subsequent quality control analyses. The original fastq data were further filtered to remove adaptors, short fragments (length < 500 bp), and low-quality reads to obtain the total clean dataset. The quality-controlled data were compared with the reference genome using minimap2 software [21]. We used Nanopolish to distinguish 5-mC in the CpG context from unmethylated cytosine, based on a hidden Markov model [22]. We used Tombo to distinguish between 5-mC in the CHH (H = A/T/C) and CHG contexts and to distinguish 6-mA from unmethylated cytosine and adenine [23].

### DNA methyltransferase and demethylase in ***B. xylophilus***

Reciprocal BlastP comparisons were conducted at the Pfam database to identify genes encoding proteins with similarities to known cytosine and adenine methyltransferases and demethylases in the PWN genome. Significant hits were defined as those satisfying the following criteria: E-value < 10^− 5^, and aligned segments covering at least 30% of the sequence length of the hit.

### Gene cloning and bioinformatics analyses of ***Bxdamt*** and ***Bxnmad***

Primers were designed based on the sequences of orthologs of genes encoding DNA-methylation enzymes in *B. xylophilus*, and were used to amplify partial segments of the potential gene (Table S2). Next, rapid amplification of cDNA ends (5′- and 3′-RACE) was performed to obtain full-length cDNAs using a SMART RACE cDNA amplification kit (Clontech, Mountain View, CA, USA) according to the manufacturer’s instructions (Table S2). The amplified products were purified using QIAquick Gel Extraction Kit (Qiagen, Hilden, Germany). Finally, the amplification products were cloned using an pEASY-Blunt Simple Cloning Kit (Transgen Biotech, Beijing, China) and sequenced.

Sequence alignment and identity analyses were performed using DNAMAN software. Open reading frames (ORFs) were identified using ORF Finder (http://www.ncbi.nlm.nih.gov/gorf/orfig.cgi). The physicochemical properties and primary structure of proteins were predicted using ExPASy (http://web.expasy.org/protparam/). Protein domains in the deduced protein sequence were identified using InterPro (http://www.ebi.ac.uk/interpro/scan.html). Predictions of protein structure and functions were made using I-TASSER (https://zhanglab.ccmb.med.umich.edu/I-TASSER ). For phylogenetic analyses, multiple sequence alignments were performed using MEGA software, with file formats converted using SeqVerter software. The phylogenetic trees were constructed using MrBayes based on Bayesian inference with the mixed model, and were displayed using FigTree software.

### Detection of ***Bxdamt*** and ***Bxnmad*** transcript levels in ***B. xylophilus***

Total RNA was extracted from samples of *B. xylophilus* using an RNeasy Mini Kit (Qiagen), and then cDNA was synthesized using the Primescript RT reagent kit with gDNA Eraser (TaKaRa, Otsu, Japan) according to the manufacturer’s protocol.

The presence and transcript levels of *Bxdamt* and *Bxnmad* were detected using qRT-PCR, with specific primers as shown in Table S2. The reactions were performed using the LightCycler 480 II system (Roche Diagnostics Ltd., Basel, Switzerland) with TB Green Premix Ex Taq (TaKaRa). The thermal cycling parameters were as follows: 95 °C for 30 s, and then 40 cycles at 95 °C for 5 s and 60 °C for 30 s. The relative transcript level of each candidate gene was calculated using the 2^−ΔΔCt^ method [24].

## Results

### Presence of methylated DNA in ***B. xylophilus***

Two types of DNA methylation, 5mC and 6mA, were detected in the PWN genome using the DNA Methylation ELISA kits. As determined using ELISA analyses, the global DNA methylation levels in the genome of PWN cultured with *B. cinerea* at 25 °C were 0.463% (5mC) and 0.025% (6mA). The UPLC-MRM-MS/MS assays detected 5mC and 6mA in *B. xylophilus* DNA, but not 5hmC and 5fC (Figure S1). As determined by UPLC-MRM-MS/MS analyses, the global DNA methylation levels in the genome of PWN cultured with *B. cinerea* at 25 °C were 0.024% (5mC) and 0.184% (6mA).

### DNA methylation profile in ***B. xylophilus***

To further analyze DNA methylation across the *B. xylophilus* genome, we used ONT methods to detect DNA methylation at the genome-wide level. After removing adapters and low-quality reads, we obtained 1,033,317 clean reads consisting of 8,962,174,105 bases, with a read N50 of 10,320 bp, corresponding to approximately 100 × genome-wide coverage (Tables S1). Approximately 78% of the clean reads were aligned to the *B. xylophilus* reference genome (ASM412881v1) and mapped. We cataloged 12,402,864 adenine sites (sequencing depth ˃ 10×coverage) and 14,151,458 cytosine sites (Sequencing depth ˃ 10× coverage). The cytosines were in three sequence contexts: CpG, 5,089,121 sites (36%); CHG, 1,981,721 sites (14%); and CHH, 7,080,616 sites (50%). These data allowed for, the methylation status of individual sites to be determined with confidence.

**Fig. 1:**
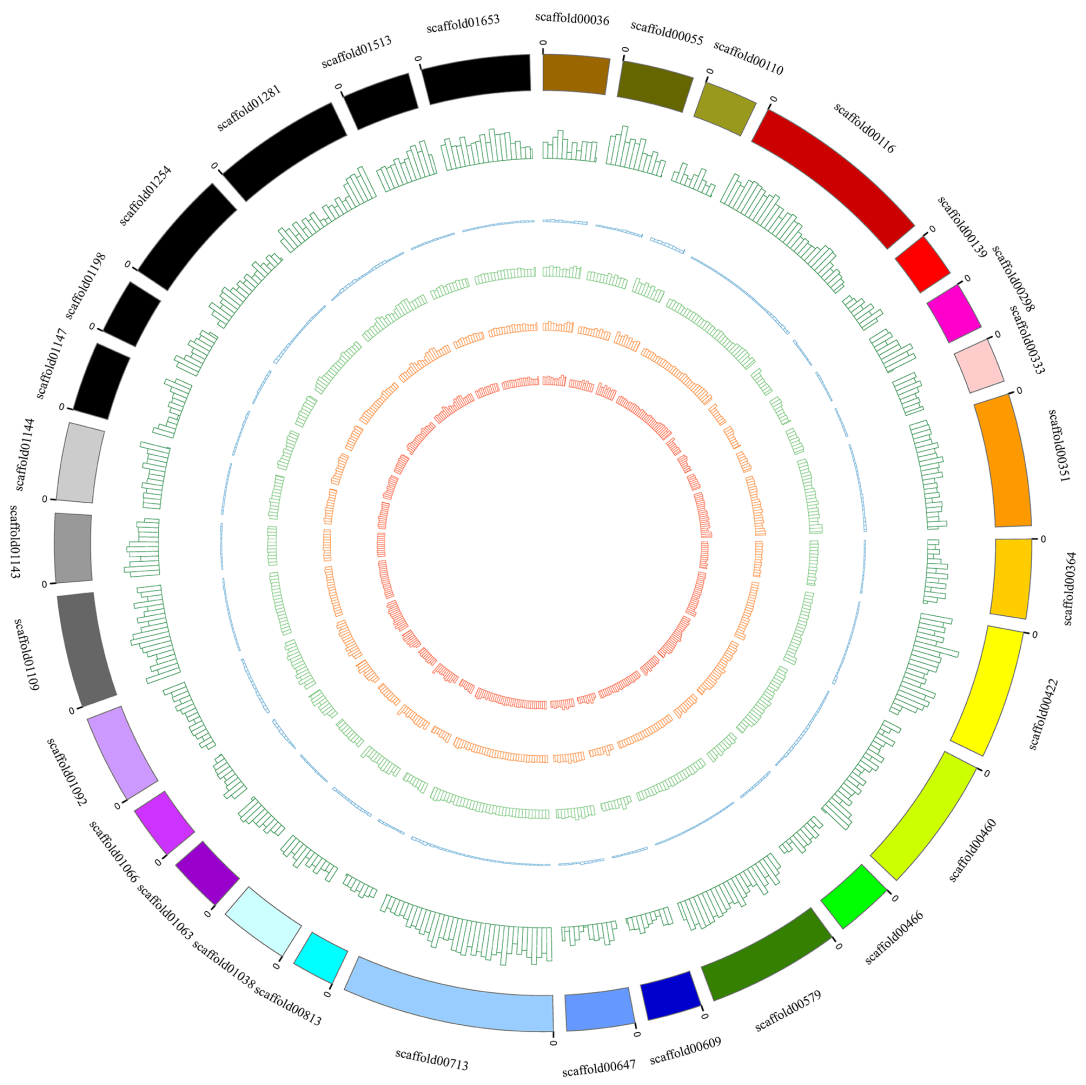
Distribution of methylation sites in pine wood nematode genome. Circos plots showed genomic methylation levels of pine wood nematode. From outside to inside, the genome scaffolds, the gene density in the window (higher means more genes there), the methylation level of CpG, CHH ˎCHG, 6mA (higher means higher methylation level, range are from 0 to 1)

The 6mA and 5mC modifications were broadly distributed across the *B. xylophilus* genome (Fig. 1). The average level of methylation in each context was calculated. Among the 12,402,864 adenine sites detected, 71% were able to be methylated and the average methylation level at each site was 0.17 (Figure S2). Among the 14,151,458 cytosine sites detected, 71% were able to be methylated and the average methylation level at each site was 0.14. Of the cytosine sites, 42.6%, 84.2%, and 87.8% of those in the CpG, CHG, and CHH (H = A, G or T) contexts, respectively, were able to be methylated, with average methylation levels of 0.039, 0.16, and 0.20, respectively (Figure S2). The lowest level of methylation was at CG sites.

### Methylation levels in regions with different annotations

Next, we examined patterns of methylation among distinct genomic elements, including genes and tandem repeats. Because DNA methylation in gene promoter regions plays an important role in the epigenetic regulation of gene transcription (Bergman and Cedar, 2013), we determined the distribution of DNA methylation around the upstream − 2 kb region of transcription start sites (TSSs) and the downstream 2 kb region of transcription termination sites (TTSs).

**Fig. 2:**
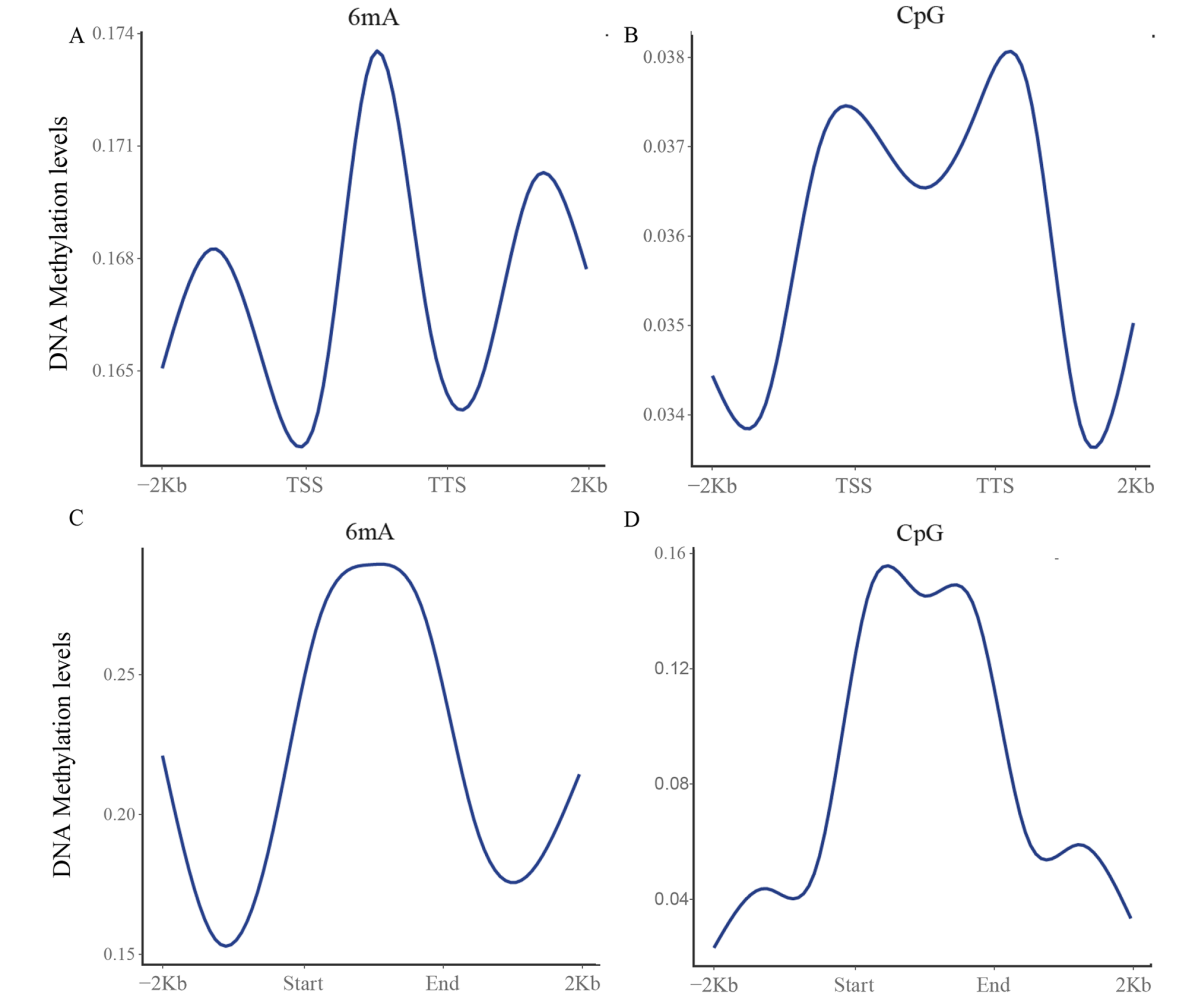
The methylation levels in regions with different annotations. (***A, C***) Methylation levels of 6mA in gene regions and repeats regions. (***B, D***) Methylation levels of CpG in gene regions and repeats regions

The CG methylation level was increased near TSSs and TTSs. In contrast, in the upstream and downstream flanking sequences, the 6mA levels rapidly increased with increasing distance from the TSSs and TTSs. Besides this feature, the levels of 6mA and methylated CpG were higher than average in gene regions, and the range of change was very low (Fig. 2A, B).

DNA and histone methylation frequently occur in DNA repeat regions to achieve epigenetic gene silencing. Different from the trends in the methylation status of gene regions, in repeat regions, 6mA and CpG methylation showed the same trend, with increased levels at the beginning of repeat regions and markedly decreased levels at the end. Besides this feature, the levels of 6mA and CpG methylation were higher than average in DNA repeat regions, and the change was very wide (Fig. 2C, D).

### DNA methyltransferase encoded in the ***B. xylophilus*** genome

To understand how DNA is methylated in *B. xylophilus*, we conducted reciprocal Blast searches to identify orthologous genes of known DNA (cytosine-5) and (adenine-6) –methyltransferases genes in the draft *B. xylophilus* genome. We did not find any orthologs of known eukaryotic cytosine methylase DNMTs, but BUX.s00333.8 showed homology to the CXXC zinc finger domain of DNMT1. We found orthologs of N6- adenine methyltransferases and N6- adenine demethylases that methylate adenine in human, mouse and *C. elegans*. Therefore, we cloned the *B. xylophilus damt* and *nmad* genes for further bioinformatics analyses.

### Sequencing and characterization of ***Bxdamt*** and ***Bxnmad***

The full-length cDNA of *Bxdamt* was 1065 bp (GeneBank accession no: MN428451) and encoded a 354 amino acid polypeptide with an estimated molecular mass of 40.5 KD and a predicted isoelectric point (pI) of 6.71. The subcellular localization of BxDAMT, as predicted by cell-PLOc 2.0, was the nucleus. The nuclear localization signal of BxDAMT was WPNKAVRRQKTY (score: 0.93128) and was located at amino acids 182–193 in the polypeptide.

**Fig. 3:**
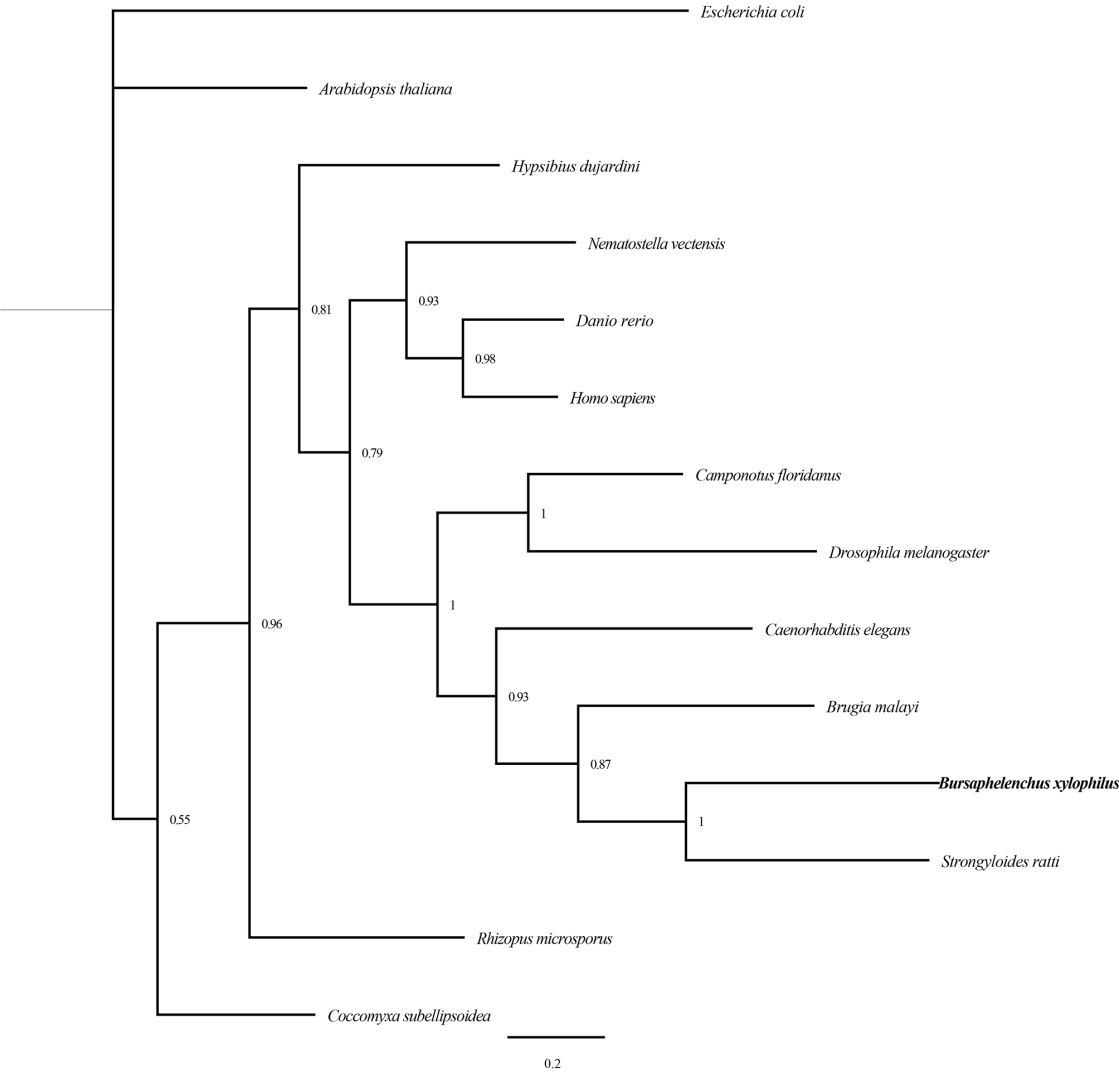
Phylogenetic relationships between BxDAMT from informative species. The phylogenetic tree was constructed with the Bayesian inference with the mixed model using MrBayes

In the InterProScan analysis, the deduced amino acid sequence of BxDAMT showed homology with N6-adenosine-methyltransferase (DAMT-1) and had typical structural features, including an MT-A 70 domain (at amino acid residues 173–337), and an S-adenosylmethionine-dependent methyltransferase domain (at amino acid residues 166–277). Multiple sequence alignment of BxDAMT proteins from different species revealed that BxDAMT was highly conserved especially in the MT-A70 domain (Figure S3). To examine the phylogenetic relationship between BxDAMT and related proteins in other species, a phylogenetic tree was constructed based on the deduced amino acid sequences from 14 species, including the model organisms *Homo sapiens*, *Danio rerio*, *Drosophila melanogaster*, and *C. elegans*. As shown in Fig. 3, the methyltransferases of most phylogenies grouped together, with posterior probability values between 0.8 and 1. The PWN BxDAMT was most closely related to those of *Strongyloides ratti* and *Brugia malayi* and clustered within a nematode clade containing *C. elegans* Damt-1. Thus, BxDAMT was identified as a putative methyltransferase in *B. xylophilus*.

The full-length cDNA of *Bxnmad* was 855 bp long (GeneBank accession no: MN428452) and encoded a 284 amino acid polypeptide with an estimated molecular mass of 33.4 KD and a predicted pI of 5.98. BxNMAD was predicted to localize to the nucleus, with the nuclear localization signal (SQSGRRKQDYGPKVNFKHKKVRP; score, 0.93117) located from amino acids 111 to 133 in the polypeptide.

In the InterProScan analysis, the deduced amino acid sequence of BxNMAD showed homology with N6-adenosine-demethylase (NMAD-1) and included typical structural features including an alpha-ketoglutarate-dependent dioxygenase alkB homolog 4 (ALKBH4) domain (residues 2–277) and a 2OG-FeII_Oxy; 2OG-Fe(II) oxygenase superfamily conserved domain (residues 83–254) (Figure S4).

**Fig. 4:**
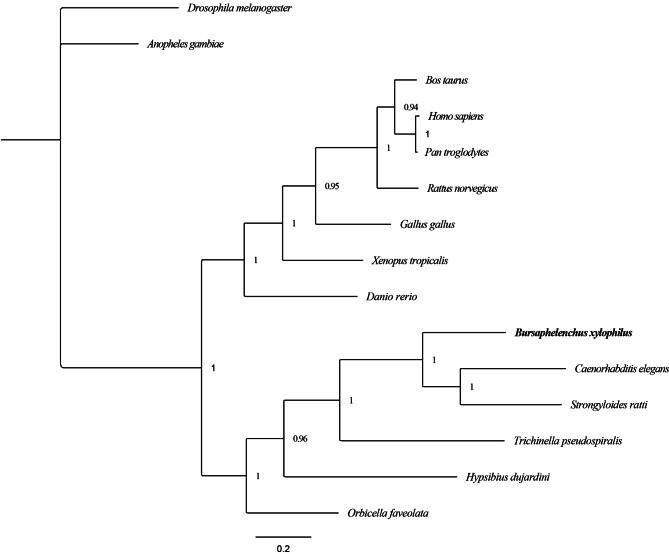
Phylogenetic relationships between BxNMAD from informative species. The phylogenetic tree was constructed with the Bayesian inference with the mixed model using MrBayes

To examine the phylogenetic relationship between BxNMAD and similar proteins in other species, a phylogenetic tree was constructed based on the deduced amino acid sequences of NMADs from 15 species, including *H. sapiens*, *D. rerio*, *D. melanogaster*, and *C. elegans*. As shown in Fig. 4, these demethylases grouped into most phylogenies with posterior probability values between 0.9 and 1. The PWN BxNMAD clustered with homologs in the nematode clade containing *S. ratti* and *C. elegans*. It was found to be highly conserved during evolution, consistent with the evolutionary relationships among these species. Thus, it was identified as a putative demethylase in *B. xylophilus*.

### Relationships between methyltransferases and DNA methylation levels

To examine the relationships between methyltransferases and methylation levels, we determined the transcript levels of *Bxdamt* and *Bxnmad* and the methylation levels in the genome of PWN at different developmental stages (egg, larval stages L2 and L4, and adult) and in response to cold temperature stress (10 °C and 4 °C).

**Fig. 5:**
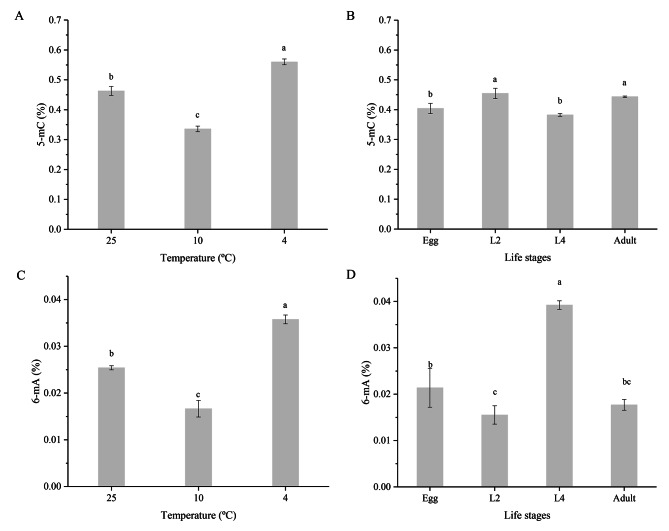
DNA methylation changes of pine wood nematodes. (**A, C**) Changes of methylation levels of pine wood nematode at different temperatures. (**B, D**) Changes of methylation levels of pine wood nematode at different life stages

The methylation level of the PWN genome was affected by temperature and differed among development stages. The 5mC and 6mA levels showed the same trends in response to cold shock (10 °C and 4 °C), being significantly downregulated at 10 °C and upregulated at 4 °C (*P* < 0.05) compared with their respective levels in the control (25 °C). However, the 5mC and 6mA levels showed different trends amongst different developmental stages. The 5mC level was higher at the L2 and adult stages than at the egg and L4 stages, while the 6mA level showed the opposite pattern (Fig. 5).

**Fig. 6:**
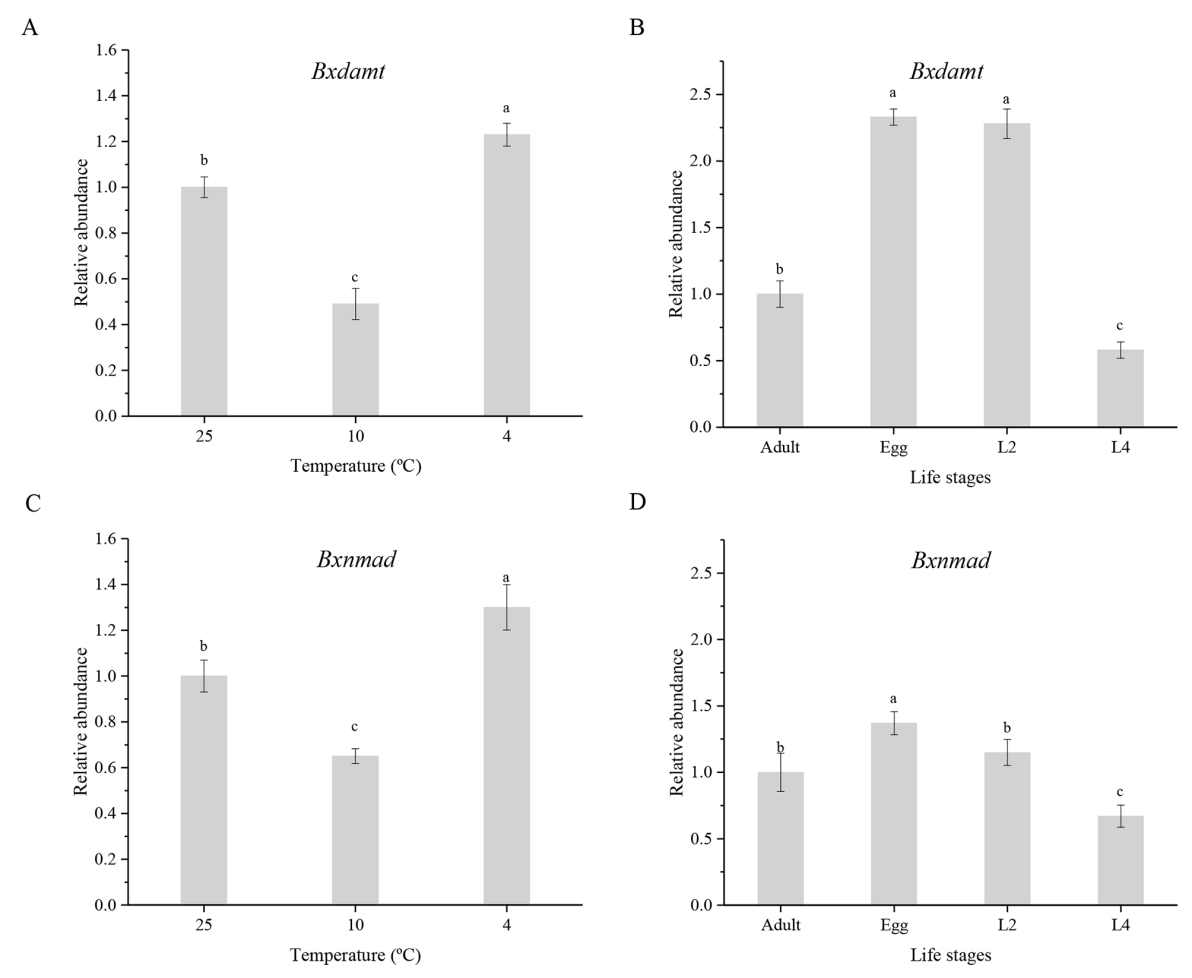
Transcript levels of *Bxdamt* and *Bxnmad* changes of pine wood nematodes (**A, C**) Changes of transcript levels of *Bxdamt* and *Bxnmad* at different temperatures. (**B, D**) Changes of transcript levels of *Bxdamt* and *Bxnmad* at different life stages

The transcript levels of *Bxdamt* and *Bxnmad* in *B. xylophilus* were affected by temperature and differed among development stages. The trends in the transcript levels of *Bxdamt* and *Bxnmad* were consistent with those of the 5mC and 6mA levels; both genes were significantly downregulated at 10 °C and upregulated at 4 °C (P < 0.05) compared with their respective levels in the controls (25 °C). Among the different developmental stages of *B. xylophilus*, eggs had higher transcript levels of *Bxdamt* and *Bxnmad* than did the L2, L4, and adult stages. The transcript levels of *Bxdamt* and *Bxnmad* gradually decreased during nematode development, reaching the lowest levels in last instar larvae. The transcript levels of both genes then recovered in the adult-stage nematodes (Fig. 6).

## Discussion

5mC is the dominant form of methylation in eukaryotes and 6mA is the most prevalent form in prokaryotes, and both types were detected in the genome of eukaryotic *B. xylophilus* by ONT and ELISA. DNA methylation types vary widely among multiple lineages. Many eukaryotic lineages have lost the 5mC modification, including the model organisms *D. melanogaster* [14] and *C. elegans* [12]. With the development of more sensitive detection techniques, 6mA has been detected in multicellular eukaryotes [10, 12, 14]. The existence of 6mA in eukaryotes is controversial because of its extremely low abundance (< 10 ppm) [25, 26]. The average methylation ratio at individual sites of cytosines and adenines detected in the PWN genome by ONT sequencing were 0.14 and 0.17, respectively, much higher than the controversial low ratios reported in other studies. Our results show that 5mC and 6mA are widely distributed in the PWN genome, although these modifications are rarely found in other multicellular eukaryotes and this is the first report of their presence in nematodes [17, 19]. Our findings also show that 5mC and 6mA are environmentally and developmentally regulated in *B. xylophilus*. These findings may be of great significance in terms of the invasiveness and evolution of PWN and for further studies on the evolution of DNA methylation pathways in different species.

DNA methylation is the process by which a methyl group of S-adenosyl methionine (SAM or AdoMet) is transferred to a specific base by a DNA methyltransferase. DNA methyltransferases, as well as the types and levels of DNA methylation, vary widely across lineages. In general, there are two types of DNA methylation: 5mC, which is catalyzed by DNA methyltransferases (DNMTs), and 6mA, which is catalyzed by 6mA methyltransferases.

All of the nematode DNMTs identified to date are homologs of DNMT1, DNMT2, or DNMT3 [27]. DNMT2, which predominantly methylates tRNA, is the most widespread among nematodes, but has been lost from some nematode lineages. DNMT1 and DNMT3, which are maintenance methyltransferases that catalyze *de novo* methylation, have been lost from many nematode lineages [13]. Research on the relationship between cytosine methyltransferases (DNMTs) and cytosine methylation found that 5mC was clearly detectable in the species containing DNMT1 or DNMT3, but not in species lacking DNMTs, such as *C. elegans* and *Caenorhabditis briggsae* [13]. However, we detected 5mC in PWN, even though we did not detect any orthologous genes to known cytosine methylase DNMT genes in the *B. xylophilus* genome. In another study on the evolution of DNA methylation, DNMTs were not detected in *B. xylophilus* using a different homology search strategy [13]. One reason for this may be the lack of a high-quality complete genome assembly. Another possible reason is that DNA methylation pathways have evolved rapidly in multiple lineages, so that some lineages of nematodes have DNMTs without typical DNMT domains. Studies have shown that cytosine DNMTs are frequently absent from multiple lineages. For example, in nematodes, some DNMTs have been detected in the early branching clades I, II, and III, but not in clades IV (*B. xylophilus*) and V (*C. elegans*) [13, 28].

The putative MTase responsible for DNA 6mA is METTL4, which is a homologous protein of MT-A70 family eukaryotic MTases in mammals [10]. BxDAMT is homologous to the identified 6mA methyltransferase DAMT-1 (*C. elegans*), METTL4 (*D. melanogaster*), and TAMT-1 (*Tetrahymena thermophila*) [29], and has a common domain, MT-A70, which is highly conserved. ALKBH1 and ALKBH4 have also been reported to function as a demethylase of DNA 6mA [10]. BxNMAD belongs to the AlkB family of dioxygenases that have been recruited as 6mA demethylases, which contain the conserved Fe^2+^ ion and a 2-oxo-glutarate-dependent dioxygenase domain. BxDAMT is homologous to the identified 6mA demethylases NMAD-1 (*C. elegans*) and DMAD (*D. melanogaster*) [12, 14]. Our results show that the transcript levels of *Bxdamt* and *Bxnmad* were affected by environmental temperature and development, and their changes were consistent with the abundance of 5mC and 6mA in response to cold temperature stress. Among the different developmental stages, eggs had higher transcript levels of *Bxdamt* and *Bxnmad* than did the newly hatched larvae, last instar larvae, and adult stages, consistent with tissue and organ differentiation during nematode development. Thus, the proteins encoded by *Bxdamt* and *Bxnmad* may regulate the methylation levels in the PWN genome.

In bacteria, 6mA is the main type of DNA methylation and is a component of the restriction-modification system that discriminates between the host genome and invader DNA. Recent studies have revealed that 6mA also exists in eukaryotic genomes. Thus, 6mA has potential epigenetic roles across diverse eukaryotes that are distinct from those of the well-studied 5mC modification [17, 19].

The distribution patterns of DNA methylation vary widely among species, and may be indicative of distinct genomic functions across diverse phylogenetic groups [19]. We found that 5mC and 6mA were widely distributed across the *B. xylophilus* genome, and the methylation levels were higher in repeat regions and lower in gene regions, compared with the average. In repeat regions, DNA modification is associated with transcriptional reactivation as well as increased mobilization of transposable elements. Methylation in gene regions may play an important role in epigenetic regulation of gene transcription (Bergman and Cedar, 2013). Methylated cytosines occur overwhelmingly at CpG dinucleotide sites in mammalian genomes, but at both CpG and non-CpG sites in plant and fungi genomes [30, 31]. Our results show that in PWN, cytosines are methylated in CpG, CHG, and CHH (H = A, G or T) contexts. In nematodes, the methylation levels in each sequence context (CpG, CHG, and CHH) may be related to the developmental stage [11]. The highest frequency of methylated cytosines was in CG regions in older larvae but in CHH regions in newborn larvae. Interestingly, we detected opposite trends in the frequencies of 6mA and methylated CpG in gene regions, and a negative correlation between 5mC and 6mA levels at different developmental stages. Therefore, the 6mA and 5mC modifications in the genome may play different functions in PWN. Further studies are required to explore the roles of 6mA and 5mC in the environmental adaptation of PWN, and their evolutionary significance.

## Conclusions

In this study, we detected the extensive presence of 5mC and 6mA in the *B. xylophilus* genome, with low methylation levels at most positions, suggesting that 5mC and 6mA are two important epigenetic markers in this invasive nematode. The 5mC and 6mA modifications appeared to occupy distinct regions of the genome, suggesting that they have distinct genomic functions. The changes in the abundance of 5mC and 6mA showed the same trends in response to temperature change, but opposite trends during development. Bioinformatics analyses showed that BxDAMT and BxNMAD have typical characteristics of a methylase and demethylase, respectively, and are conserved among species. Thus, these two proteins are highly likely to mediate DNA methylation in PWN. These findings shed light on the epigenetic modifications present in the genome of PWN, and will improve our understanding of its invasiveness and evolution.

### Electronic supplementary material

Below is the link to the electronic supplementary material.

Supplementary Material 1

## Data Availability

The datasets generated during the current study are available in the GeneBank repository, accession no: MN428451 and MN428452.
