# Supplementary material for: DNA methylation on C5-Cytosine and N6-Adenine in the Bursaphelenchus xylophilus genome
Source: BMC Genomics. 2023 Nov 7;24:671. doi: 10.1186/s12864-023-09783-7 (PMC10631105; doi:10.1186/s12864-023-09783-7)
Supplement: Supplementary file 1 — Supplementary Material 1 [file 12864_2023_9783_MOESM1_ESM.docx]

**Supplementary Information**

**
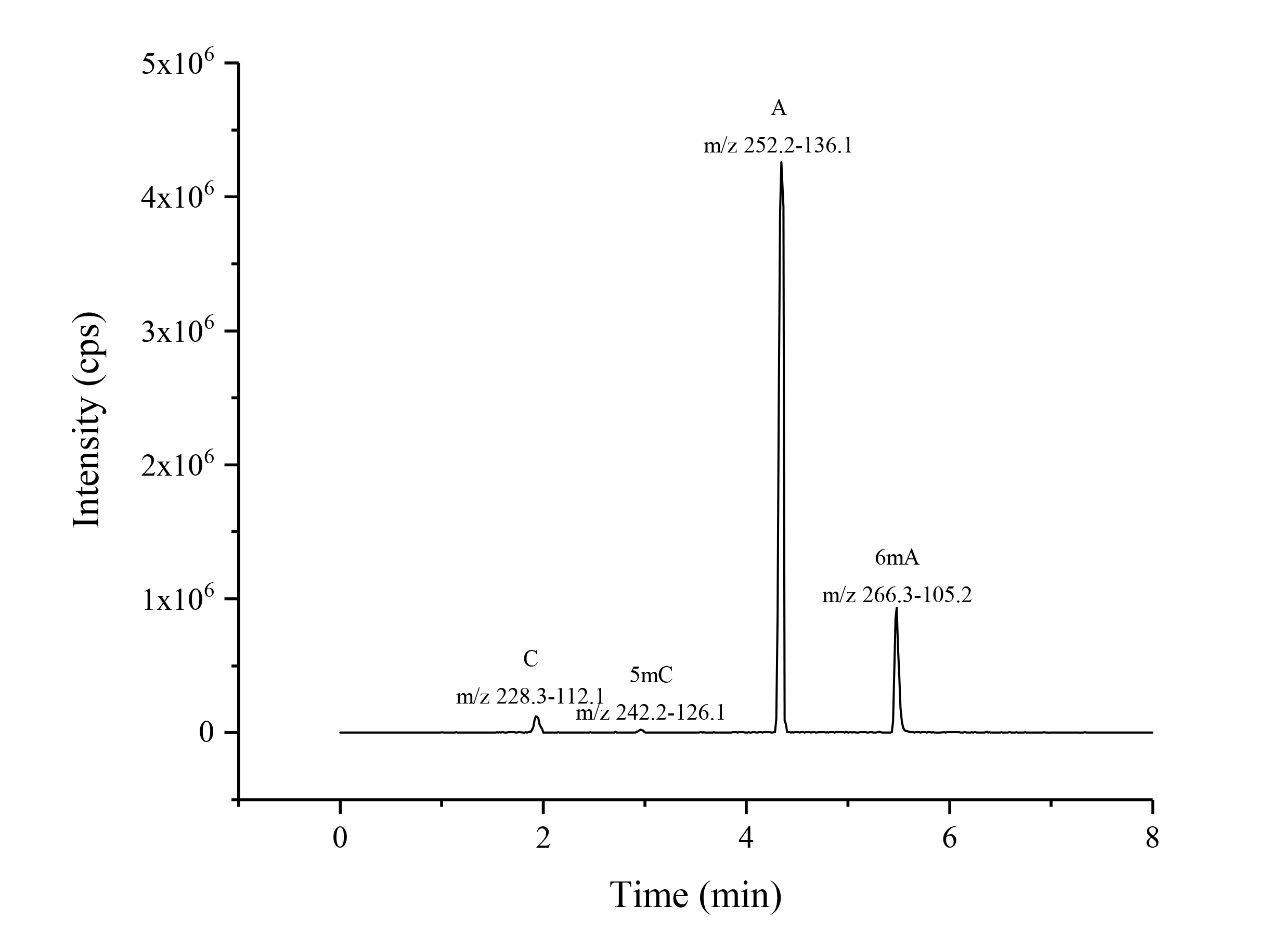
**

**Figure S1 UPLC-MRM-MS/MS chromatograms of 5mC and 6mA in pine wood nematode**


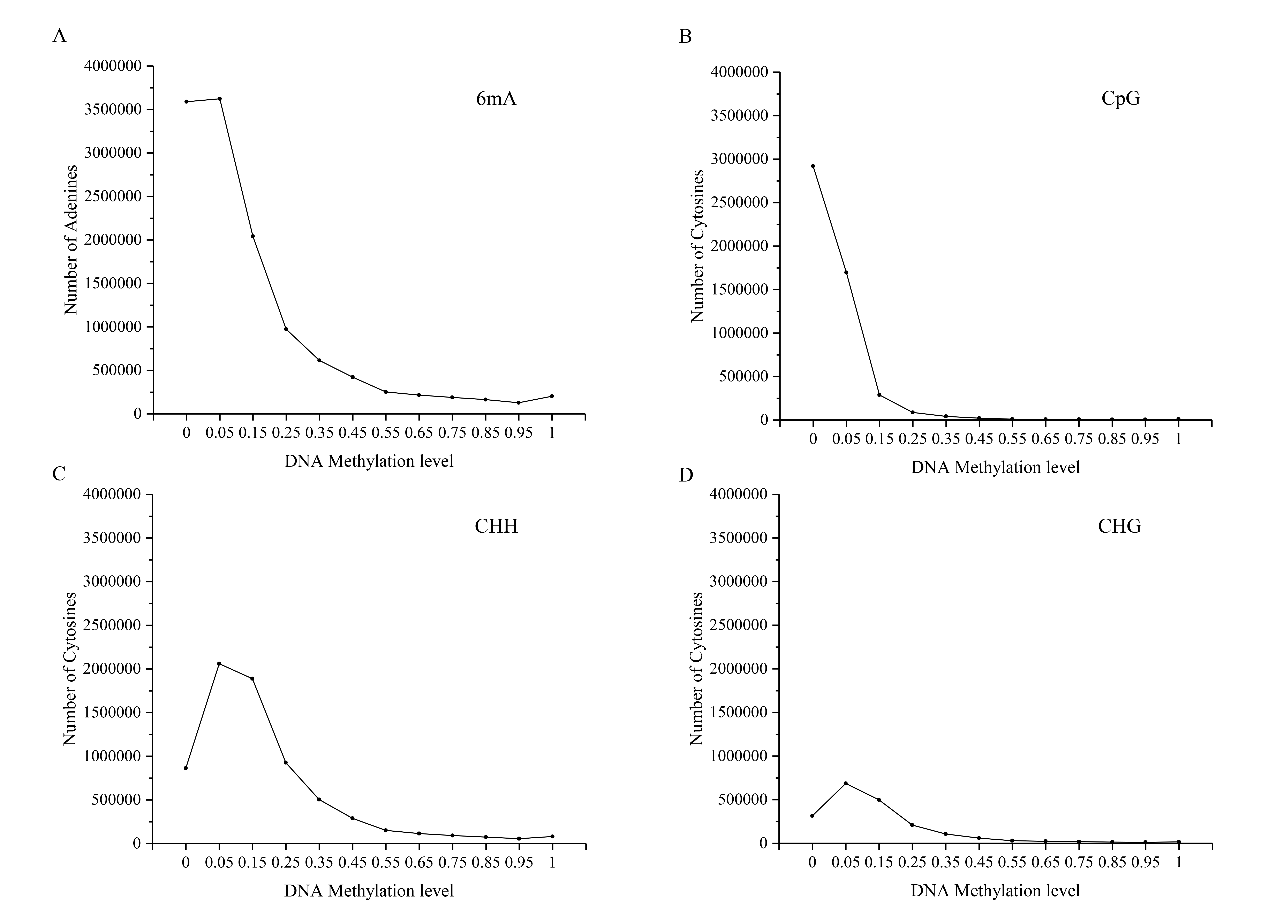


**Figure S2 Distribution of DNA methylation levels in pine wood nematode**

The X-axis shows the level of methylation (methyl/cover). methyl: The methylatd reads of the site; cover: The methylatd and unmethylated reads of the site. Except 0 and 1, other values on the X-axis represent interval values, such as 0.05 (0<methylation level≤0.1), 0.15(0.1<methylation level≤0.2) and so on.

**
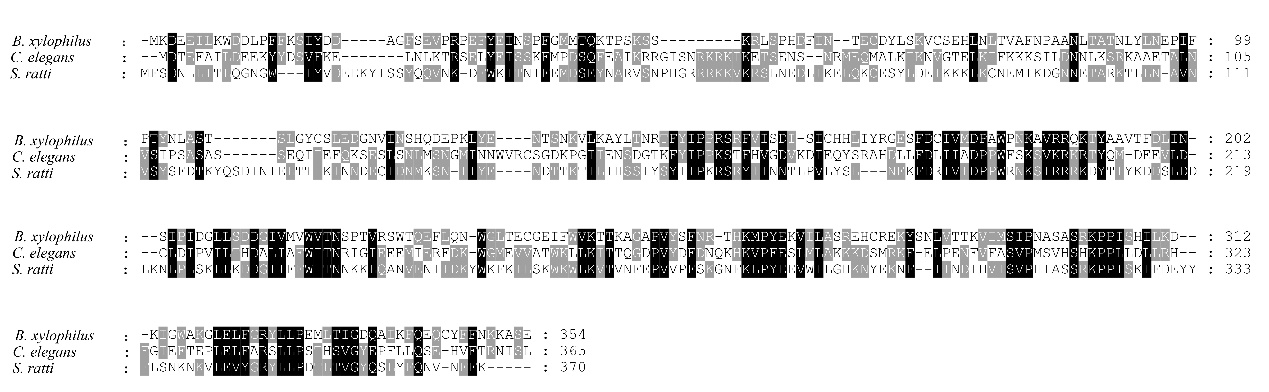
**

**Figure S3 Multiple alignment of BxDAMT from *B. xylophilus* and its homologues in other nematodes**


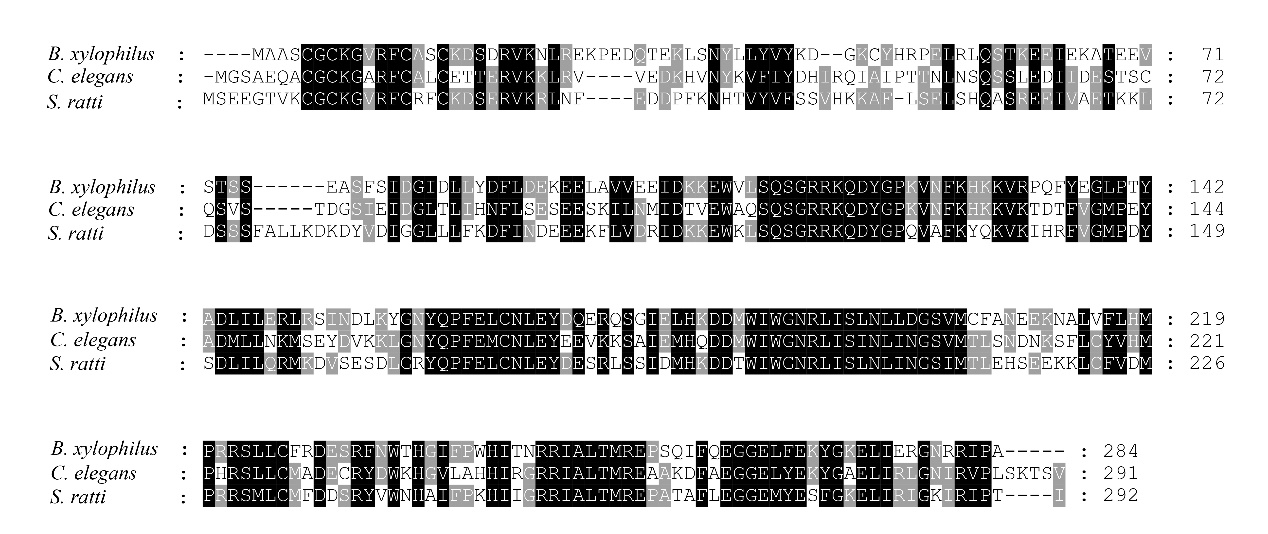


**Figure S4 Multiple alignment of BxNMAD from *B. xylophilus* and its homologues in other nematodes**

**Table S1 Summary sequencing statistics**

| Species | Number of Reads | Bases Sequenced | Read N50 | Mean Read Length | Avg Reads Quality |
| --- | --- | --- | --- | --- | --- |
| *B. xylophilus* | 1,033,317 | 8,962,174,105 | 10,320 | 8,673 | 10.06 |

**Table S2 Primers used in the experiments**

| Number | Primer Name | Primer Sequences (5’-3’) |
| --- | --- | --- |
| 1 | *Bxdamt* F | CCGATGATGGAATTGTTATGG |
| 2 | *Bxdamt* R | GGTTGTCACGAGATTACTGT |
| 3 | *Bxnmad* F | TCGGCGGTTA GTTATATGC |
| 4 | *Bxnmad* R | TGA TGG ATCT GTG ATG TGTT |
| 5 | dT | CCAGTGAGCAGAGTGACGAGGACTCGAGCTCAAGCTTTTTTTTTTTTTTTTT |
| 6 | d0 | CCAGTGAGCAGAGTGACG |
| 7 | d1 | GAGGACTCGAGCTCAAGC |
| 8 | *Bxdamt*- d0 | CCGATGATGGAATTGTTATGG |
| 9 | *Bxdamt* - d1 | ATTGACGGAATGCGGAGA |
| 10 | *Bxnmad* - d0 | TGCTGTGGTTGAGGAGAT |
| 11 | *Bxnmad* - d1 | TCTCAGTCTCAACCTCCTT |
| 12 | dG | AAGCAGTGGTATCAACGCAGAGTACGCGGG |
| 13 | UPM | AAGCAGTGGTATCAACGCAGAGT |
| 14 | *Bxdamt* - d2 | GGTTGTCACGAGATTACTGT |
| 15 | *Bxdamt* - d3 | GCATCTTATGTGTCCGATTG |
| 16 | *Bxnmad* - d2 | GGCATTCTTCTCTTCATTGG |
| 17 | *Bxnmad* - d3 | AAGGAGGTTGAGACTGAGA |
| 18 | *Bxdamt*-q F | ATGTCTATCCCCAACGCAAG |
| 19 | *Bxdamt*-q R | TCGCCTATCGTCAGCA TTC |
| 20 | *Bxnmad*-q F | GGATCTGTGATGTGTTTTGCC |
| 21 | *Bxnmad*-q R | GAACCGACTCTCATCTCTGAAG |

Primer (1-4) for amplification of *Bxdamt* and *Bxnmad* gene fragment. Primer (5-17) for amplification of Rapid Amplification of cDNA Ends. Primer (18-21) for amplification of qRT-PCR.
